# Si Inhibited Osteoclastogenesis: The Role of Fe and the Fenton Reaction

**DOI:** 10.1002/adhm.202501086

**Published:** 2025-06-05

**Authors:** Yutong Li, Adriana‐Monica Radu, Azadeh Rezaei, Joel Turner, Kaveh Shakib, Akiko Obata, Toshihiro Kasuga, Gavin Jell

**Affiliations:** ^1^ Division of Surgery & Interventional Science University College London London NW32QG UK; ^2^ Division of Advanced Ceramics Nagoya Institute of Technology Gokiso‐cho, Showa‐ku Nagoya 466–8555 Japan

**Keywords:** bioactive glass, fenton reaction, osteoclasts, reactive oxygen species (ROS), silicate

## Abstract

Soluble silicate species (Si) released from bioceramics have been reported to enhance bone regeneration and regulate bone remodeling, but their cellular mechanisms remain unclear. Here, we show that Si inhibited osteoclast (OC) formation and resorption (as determined by OC number, TRAP‐5b expression, and volumetric resorption) in a concentration‐dependent manner, in both primary OCs and an osteoclastic subclone of the RAW264.7 cell‐line (p<0.001). We propose that Si‑mediated inhibition may be mediated via reduced reactive oxygen species (ROS) availability, due to iron‑silicate (Fe–O–Si) interactions. Biochemical assays demonstrated that Si decreased the availability of ROS, and decreased intracellular ROS concentrations in OCs, but only in the presence of Fe. Furthermore, Si‐inhibited OC formation is restored by the addition of 10µM Fe (P<0.001). Fe‐Si interactions were demonstrated by a reduction in Si and Fe concentrations (P<0.001) in solution (ICP‐OES) and by Si─O─Fe bonds observed in precipitates (ATR‐FTIR). These results suggest that that Si may inhibit OC activity by regulation of the Fenton reaction. Understanding the intracellular mechanisms of how Si interacts with OCs allows the development of new Si‐releasing materials (e.g., bioactive glasses or bioceramics) with controlled release of Si to regulate osteoclastogenesis.

## Graphical abstract

Silicate (Si) inhibition of osteoclastogenesis, is mediated by Fe. Si chemical interactions with Fe inhibit the Fenton reaction and intercellular ROS availability. This reduction in ROS availability inhibits osteoclastogenesis. The addition of Fe, in Si‐inhibited osteoclast cultures, restores the Fenton reaction, and osteoclastogenesis.

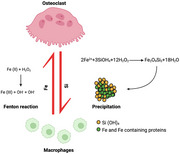

## 1 Introduction

Since the invention of bioactive glasses (BGs) by Larry Hench in 1969,^[^ ^1^ ^]^ considerable research activity has attempted to design biomaterials that release soluble silicate species (Si ions) at specific concentrations to promote bone regeneration. The dissolution products from Si‐based BG have been reported to stimulate bone regeneration both in vitro^[^ ^2^ ^]^ and in vivo.^[^ ^3^ ^]^ Of the ions released by BGs, the osteogenic properties of soluble Si ions have received considerable attention, possibly because of dietary studies in which soluble Si deprivation inhibits normal bone development,^[^ ^4^ ^]^ and where Si dietary supplements increase bone mineral density^[^ ^5^ ^]^ and can help prevent osteopenia.^[^ ^6^ ^]^ A number of studies have investigated the role of soluble Si ions on osteoblasts^[^ ^7^, ^8^, ^9^ ^]^ and have shown that soluble Si ions can increase osteoblast proliferation and upregulate osteogenic‐specific genes, e.g., bone morphogenic proteins (BMPs) and alkaline phosphatase (ALP),^[^ ^8^, ^9^ ^]^ and a number of soluble (such as vitamin D3) and non‐soluble proteins involved in bone formation (e.g., collagen type I).^[^ ^10^, ^11^ ^]^ Less, however, is understood about the effect of Si ions on OCs and bone remodeling.

Both OCs and osteoblasts are vital for healthy bone remodeling and repair following trauma. The impairment of OC function can lead to low bone turnover and can contribute to bone diseases.^[^ ^12^ ^]^ Furthermore, the chemical inhibition of OC function (for example, with bisphosphonates) has been reported to increase bone mineral density,^[^ ^13^ ^]^ leading to stiffer, more brittle, and crystalline bones,^[^ ^14^ ^]^ which have an increased risk of fracture,^[^ ^15^ ^]^ and reduced fracture healing rates.^[^ ^16^ ^]^

Of the few studies investigating the role of soluble Si on OC formation and function, it has been reported that Si inhibits osteoclastogenesis and resorption in cultures using human CD14+ monocytes,^[^ ^17^ ^]^ bone marrow‐derived cells,^[^ ^18^ ^]^ and macrophage‐like (RAW264.7) cells.^[^ ^18^, ^19^ ^]^ It has also been reported that Si (10 µM) increases gene expression of the osteoclastogenesis inhibitory factor osteoprotegerin (OPG) and inhibits the gene expression of RANKL, a vital osteoclastogenesis factor in bone‐like SaOs‐2 cells.^[^ ^19^ ^]^ It is, however, not clearly understood if Si ions influence pre/post‐transcriptional events, or how Si regulates gene expression.^[^ ^9^ ^]^ Given the central role of oxidative signaling in bone remodeling, and the known interactions between iron, reactive oxygen species (ROS), and bone‐resorbing enzymes, we hypothesized that Si may regulate osteoclastogenesis through modulation of intracellular ROS availability. This paper explores a possible link between soluble Si, Fe, and the availability of intracellular ROS on bone remodeling.

ROS plays an important role in bone remodeling through involvement in a number of intracellular signaling pathways that are critical in osteoblast‐osteoclast talk and osteoclastogenesis, including the regulation of MAPK, NFkB, RANK, and RANK ligand (RANKL) expression.^[^ ^20^, ^21^, ^22^ ^]^ ROS also plays a vital role in the function of tartrate‐resistant acid phosphatase (TRAP) and thereby bone resorption. A reduction in ROS availability may therefore inhibit osteoclastogenesis and bone resorption. TRAP is a Fe‐containing enzyme found in OC vesicles that reacts with hydrogen peroxide (via the Fenton reaction) to produce ROS to facilitate the destruction of collagen I fragments when endocytosed.^[^ ^23^ ^]^ A reduction in ROS availability may therefore inhibit osteoblast‐osteoclast regulation, osteoclastogenesis, and bone resorption.

Silicate may modulate bone remodeling by reducing the availability of ROS within OCs. Kakabadse and Dewsnap,^[^ ^24^ ^]^ when investigating the chemistry of silicic acid in solution, described the potential antioxidant capacity of Si(OH)_4_ and the reaction between sodium silicate (Na_2_SiO_3_) and H_2_O_2_.^[^ ^24^ ^]^ It has also been suggested that Si reduces Fe^2+^ and thereby decreases free radical formation (via the Fenton reaction) in environmentally friendly wastewater treatment,^[^ ^25^ ^]^ and paper making.^[^ ^20^ ^]^ Silicate may, therefore, reduce ROS availability, and thereby inhibit osteoclastogenesis and, or OC activity, via the inhibition of the Fenton reaction. Furthermore, to demonstrate the in vivo importance of Fe in bone metabolism, the use of the iron chelator desferrioxamine (DFO) has been shown to reduce osteoclastogenesis.^[^ ^26^ ^]^ Conversely, patients with systemic iron overload disorders such as haemochromatosis demonstrate elevated osteoclastogenic activity and osteoporosis.^[^ ^26^ ^]^ Therapeutic soluble silicate supplementation, may, therefore, represent a novel approach to mitigate bone loss in this patient population.

A better understanding of the role of Si on OC, both in terms of the concentration and the intracellular mode of action, would allow for the development of materials with greater precision of action and control of OC inhibition (for example, in terms of duration and concentration of Si release). This may be particularly useful for targeting specific bone diseases or patients with underlying pathologies, where controlled OC regulation would be beneficial.

## 2 Results

### 2.1 The Role of Si on Osteoclast Formation

**Figure 1:**
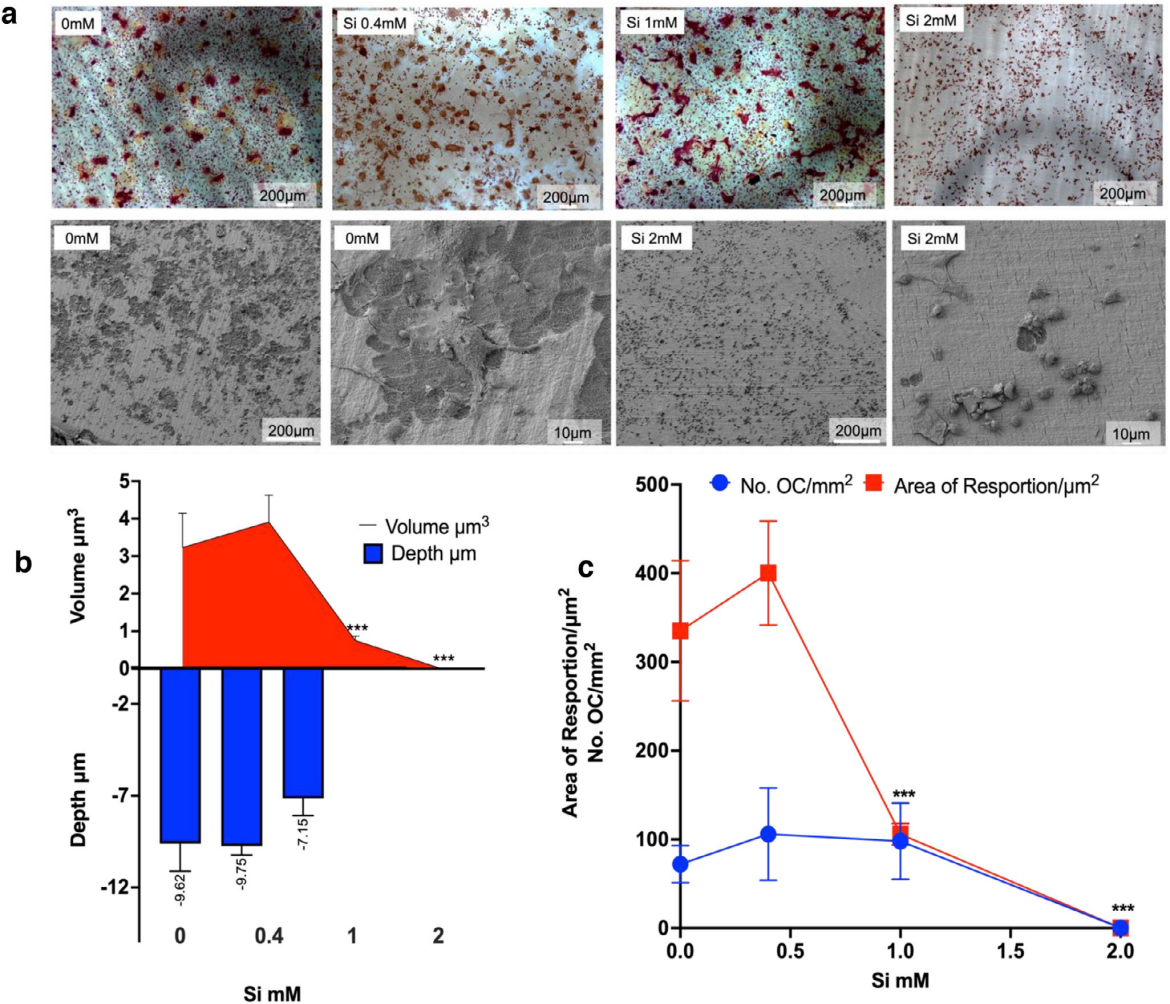
The effect of Si on primary osteoclast (OC) activity. Si inhibited OC formation and bone resorption in primary monocytes (MF‐1 mice) differentiated after 9 days of culture as observed by TRAP staining (**a**: at top) and SEM (**a**: at bottom). Quantification of the area of dentin resorbed by primary OCs revealed a Si concentration‐dependent effect on both the depth and volume of the resorption pit (b). Si over 1 mM completely inhibited OC resorption, formation, and area of resorption <0.001 (b‐c). There was no difference in the number of OCs (3 or more nuclei) at concentrations below 2 mM Si (c) (n>8) for each experimental condition, the resorption pits on discs were determined by Zygo interferometer, MetroPro, and ImageJ software; error bars represent standard deviation; p<0.001).

**Figure 2:**
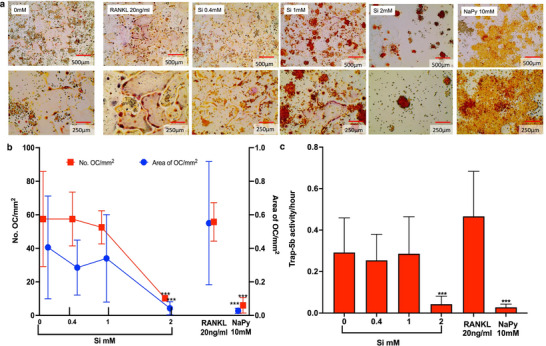
Si inhibition of subclone of RAW264.7 osteoclast (OC) formation and activity. Si 2 mM inhibited OC formation as determined by TRAP staining after 5 days of culture (a), OC quantification (number of cells with > 3 nucleus) (b), and TRAP 5b activity (c). The free radical scavenger sodium pyruvate (NaPy) [10 mM] also inhibited OC formation (***p<0.001). n>6 for each experimental condition.

**Figure 3:**
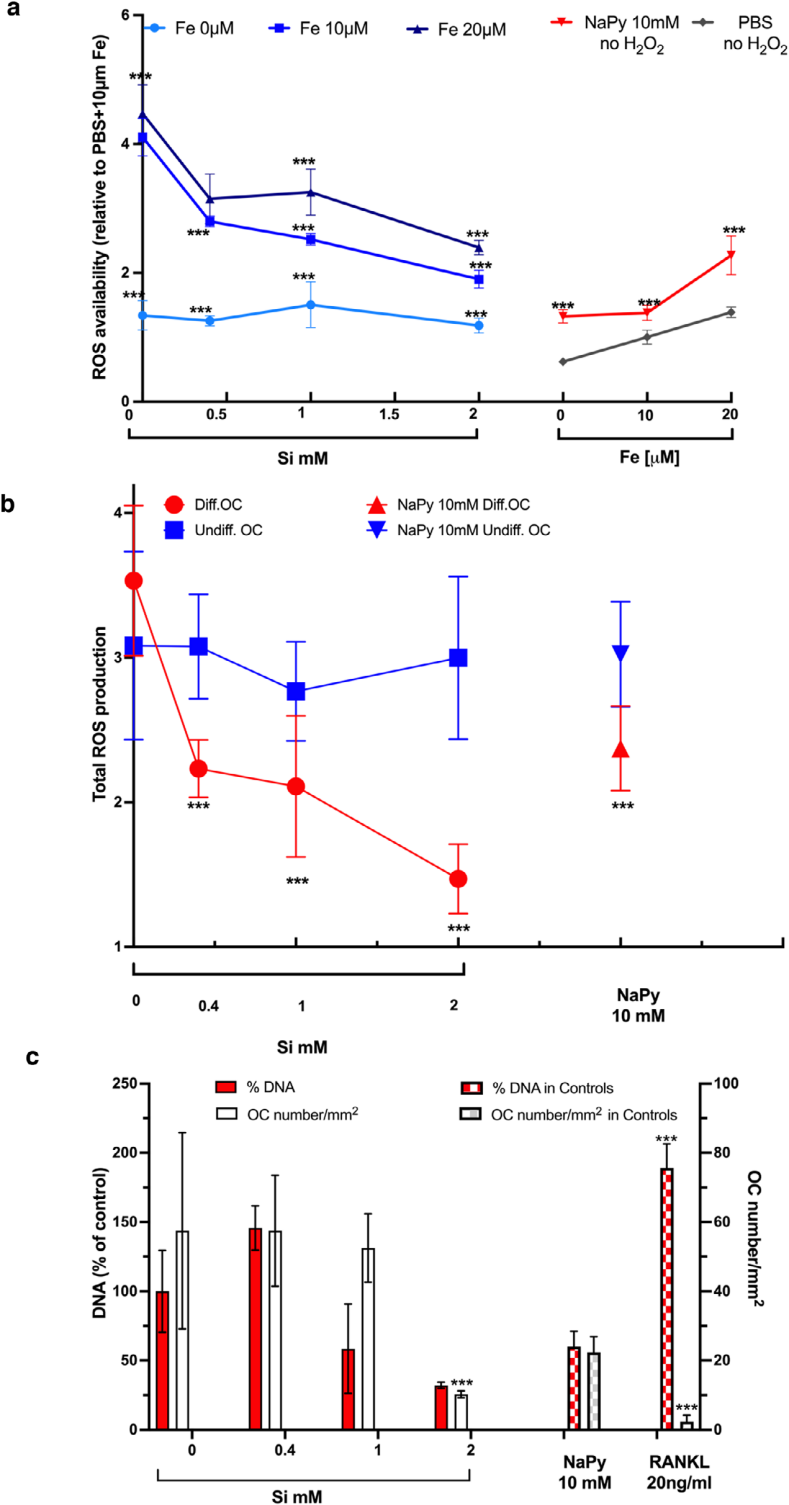
Si inhibits the Fenton reaction and reduces free radical availability. In a cell‐ free biochemical assay, Si inhibited ROS availability in a concentration‐dependent manner in the presence of FeCl_2_. Increasing [FeCl_2_] (10 to 20 µM) reduced Si‐dependent ROS inhibition. The cell free system exposed with Si contains 1 mM of H_2_O_2_. The antioxidant capacity of 2 mM Si was not significantly different to the known free radical scavenger sodium pyruvate (NaPy) (10 mM) (a). Total ROS production of Si‐treated RAW264.7 OC cultures (6 days) and in undifferentiated heterogeneous RAW264.7 cells (3 days) (b). The number of OCs formed (>3 nuclei multinucleated cells and total cells compared with 3 ng ml^−1^ RANKL control) was lower in cells treated with Si 2 mM and with the antioxidant NaPy 10 mM (c) (*** p<0.001, n>6 for each experimental conditon).

Complete inhibition of OC formation was observed in the presence of 2 mM Si in both primary OCs (**Figure** **1**) and RAW264.7 OC subclone cells (**Figure** **2**). In primary OCs, there was a Si concentration‐dependent effect on bone resorption, where 1 mM Si reduced resorption whilst complete inhibition occurred with 2 mM Si (Figure 1a–c). Concentrations of Si below 1 mM did not influence the number of primary OCs formed or the amount of resorption (Figure 1c). SEM and microscopy revealed that the addition of Si did not reduce the total number of primary cells (numerous small undifferentiated macrophages are present) but rather inhibit osteoclastogenesis (Figure 1a). The culture of OC‐like cells with the ROS scavenger NaPy (10 mM) also resulted in complete inhibition of OC‐like cell formation (Figure 2a). There were, however, differences between the effect of 10 mM NaPy and 2 mM Si on osteoclastogenesis, where NaPy appeared to increase the number of single nuclei cells present (undifferentiated osteoclasts) whilst 2 mM Si reduced the total cell number (Figures 2 and 3c). This suggests that there are differences in the mode of action of Si and NaPy on osteoclastogenesis.

It's worth noting that OC morphology and dentin resorption capacity varied between the OC cell lines (Figures 1 and 2). The RAW 264.7OC subclone cells (in both 3 and 20 ng ml^−1^ RANKL‐treated controls) differentiated into more “pancake‐like” OCs (Figure 2a), compared to the rounder, smaller OCs produced by primary cells (Figure 2a). Whilst SEM revealed that OC derived from RAW 264.7 is capable of dentin resorption (Figure S1, Supporting Information) the resorption pits were shallower than those created by primary OCs. Volume quantification of resorption by RAW 264.7 OCs was below the resolution limits of the interferometer system and therefore not possible to quantify.

### 2.2 Si Inhibits ROS in the Presence of Fe

Si inhibited ROS availability (in an H_2_O_2_ biochemical assay) in a concentration‐dependent manner, but only in the presence of Fe (Figure **3**a). Si (2 mM) reduced H_2_O_2_ (1 mM) ROS availability by more than 50%, when in the presence of Fe (10 mM), and to similar levels as the free radical scavenger, NaPy (10 mM) (Figure 3a). Higher concentrations of Si (above 2 mM) did not cause further reductions in ROS levels (data not shown). The addition of more FeCl_2_ (from 10 – 20 mM) increased ROS availability (>30%), suggesting a competitive relation between Fe and Si, with increasing Fe diminishing the effect of Si on ROS availability. Si may therefore act as an iron chelator and inhibit ROS generation via the Fenton reaction.

In vitro experiments produced similar results, where Si reduced ROS availability in OC‐like cells (after 6‐day culture) in a [Si] dependent manner (Figure 3b). Interestingly, this Si‐dependent ROS reduction was not observed in undifferentiated osteoclasts (Figure 3b). The availability of ROS in the 2 mM Si‐treated OCs was not significantly different from that observed by the free radical scavenger NaPy (10 mM). Si also reduced ROS availability in osteoblasts (SaOs‐2) at different concentrations compared to OCs (Figure S2, Supporting Information). Cell‐type specific effects of [Si] on ROS availability were therefore observed between OCs, macrophages (undifferentiated OCs), and osteoblasts.

### 2.3 Fe Supplementation Restores Si‐Inhibited Osteoclastogenesis

**Figure 4:**
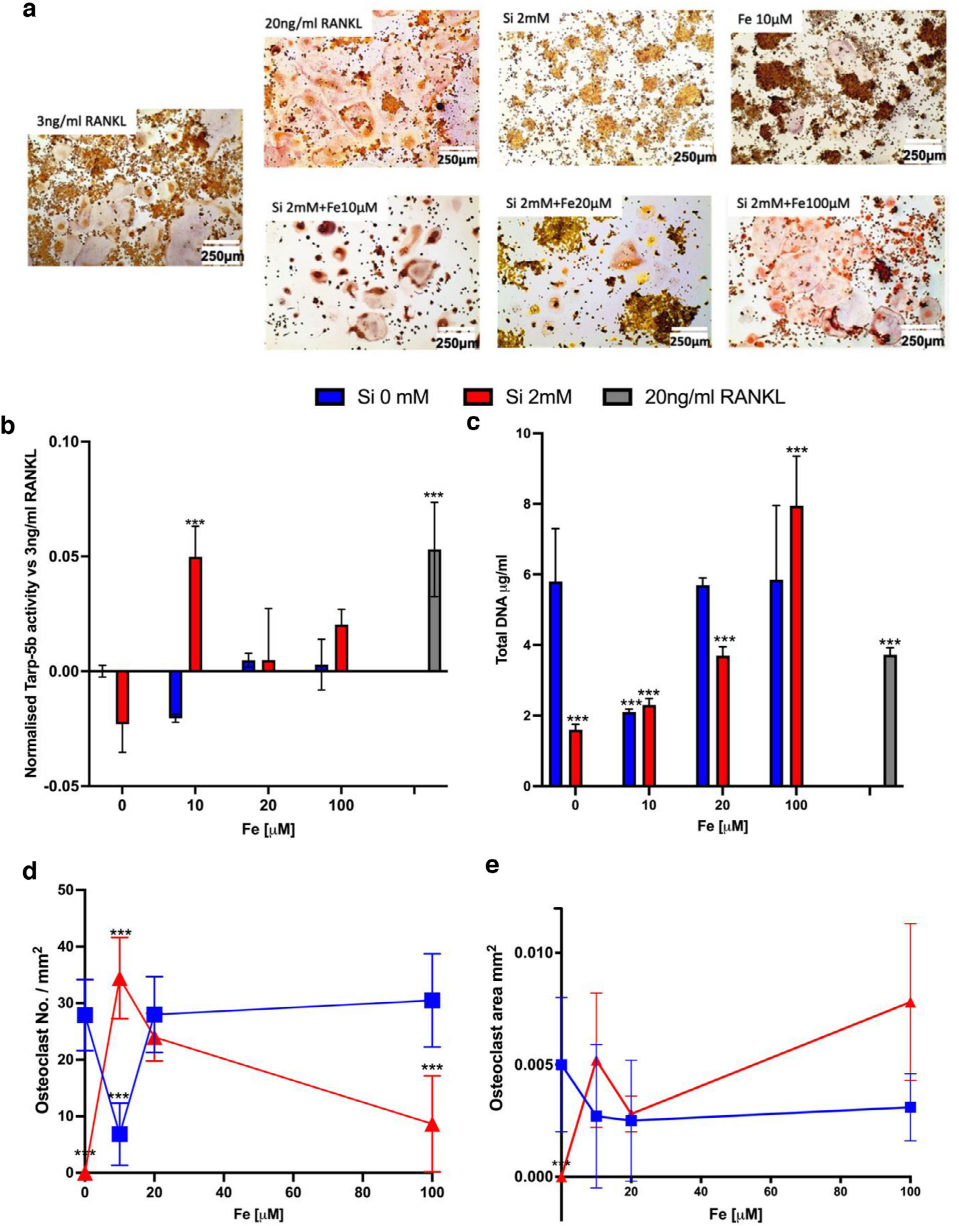
Fe restores Si inhibited osteoclast (OC) formation. The addition of FeCl_2_ (10 µM) restored Si‐inhibited OC formation as determined by TRAP staining (a), quantitative TRAP‐5b assay (displayed as normalised to 3 ng ml^−1^ RANKL control sample) (b), and OC number (as determined by a number of 3+ nuclei cells per mm^2^) (d). A Fe concentration‐dependent increase in total DNA mg/ml in both Si+ and Si‐ cultures (Fe10‐100 µM) was observed (c). There is no significant change in average OC areas in mm^2^ across all Fe concentrations (e) (***p<0.001 compared with RANKL at 3 ng ml^−1^, n>6 for each experimental condition).

The addition of soluble Fe (10 µM), restored Si‐inhibited osteoclastogenesis (as determined by TRAP‐5b staining (**Figure** **4**a), TRAP‐5b quantification (Figure 4b), and OCs number (Figure 4d)), to levels present in untreated controls. The addition of increased Fe concentrations (20–100 µM) did not further enhance osteoclastogenesis above the addition of 10 µM Fe. Furthermore, the addition of Fe, in non‐Si treated cells, did not enhance osteoclastogenesis (Figure 4a–c).

### 2.4 Si and Fe Precipitation

**Figure 5:**
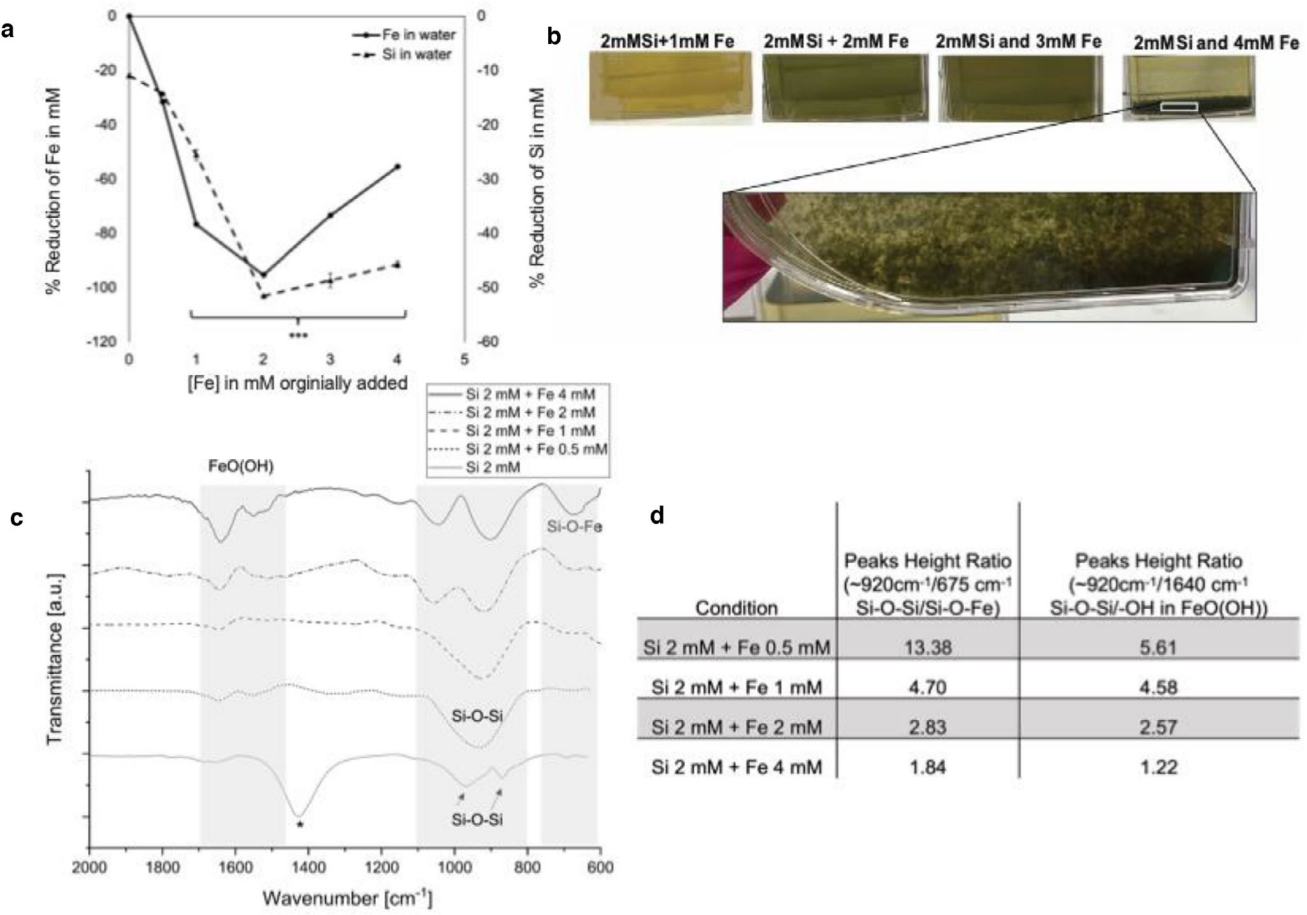
The effect of Si on Fe bioavailability. Increasing the [Fe] in 2 mM Si (FeCl2 and Na2SiO3 solutions in water) decreased the bioavailability of Fe and Si within the solute as measured by ICP, suggesting precipitation (a). On addition of 2 mM Si and 2 mM Fe there was a reduction of the soluble Fe availability by 95% and Si by 52%. Precipitation (green) was also visibly observed with increasing [Fe] (b). FTIR analysis demonstrated the presence of Fe‐Si (ferric silicate) within the precipitates (c), the shadowed areas corresponding to the characteristic spectral regions for Si‐O‐Fe and Si‐O‐Si and potentially ‐OH bonds in FeO(OH), associated IR vibrations (c). The relative peak height ratios of Si‐O‐Si to Si‐O‐Fe and Si‐O‐Si to ‐OH in FeO(OH) decreased as the Fe concentration increased with increased presence of Si‐O‐Fe with increasing [Fe] (d). *The Na‐O bond is exclusively found in the 2 mM Si sample, as it was not possible to wash after freeze‐drying due to solubility. In contrast, the remaining freeze‐dried samples consisted of both insoluble and soluble compounds (such as Na salts), which were removed during washing (c).

We have reported that Si reduces ROS availability in the presence of Fe (Figure 3) and have demonstrated that Si‐inhibited osteoclastogenesis can be restored with the addition of Fe (Figure 4), suggesting a chemical interaction between Fe and Si. To corroborate this finding, the potential precipitation of Si and Fe was investigated. In water solutions containing 2 mM Si (NaSiO_3_) and FeCl_2_ (0.5 to 4 mM), a reduction of Fe and Si ion levels in solution was observed (as determined by ICP, Figure **5**a), together with [Fe] dependent precipitation (Figure 5b). The ratio of Fe to Si influenced ion availability in solution, with a 1:1 ratio (2 mM Fe and 2 mM Si) resulting in the largest decrease in Fe and Si (a 95% reduction in Fe ions and a 52% reduction in Si ions, Figure 5a). Furthermore, at Fe to Si ratios of 1:1 ratio and above, the color of the precipitate changed from brown to green (Figure 5b), suggesting a change in Fe oxidation state. Assessment of chemical bonds in isolated precipitates by ATR‐FTIR showed peaks at ≈675 cm^−1^ corresponding to the Si‐O‐Fe symmetric stretching^[^ ^27^, ^28^ ^]^ and the Si‐O‐Fe peak intensity increased with increasing Fe concentration (Figure 5c).

Furthermore, in the 2 mM Si sample, symmetric and asymmetric characteristic silica vibrations were observed at 970 cm^−1^ and 870 cm^−1^, indicative of Si‐O‐Si stretching. Upon addition of Fe, an alteration in the Si‐O‐Si vibration pattern is observed, manifesting as a broad singular band spanning 800 to 1080 cm^−1^ for 0.5 mM and 1 mM Fe, whereas individual peaks shifted toward higher wavenumbers for Fe concentrations of 2, 3, and 4 mM, with maximum intensities at 920–1065 cm^−1^, 920–1039 cm^−1^ and 904–1052 cm^−1^ (Figure 5c). Additionally, the complexity of these peaks, characterized by shoulders and the presence of smaller peaks (≈1240 cm^−1^), suggests the formation of long silicate chains.

While the height of the Si‐O‐Si peak ≈920 cm^−1^ remained almost constant, the ratio of the Si‐O‐Si to Si‐O‐Fe peak height decreased, as Fe concentration increased (Figure 5c,d). This suggests that more Si‐O‐Fe bonds were formed as the Fe concentration increased. The ≈1530cm⁻¹ peak increased in height at higher Fe concentrations (Figure 5c) and is likely associated with the stretching vibrations of hydroxyl groups in iron oxyhydroxides (FeO(OH)).^[^ ^29^ ^]^ The adjacent peak at ≈1640 cm⁻¹ may be attributed to ‐OH bending vibrations of adsorbed water in FeO(OH). The ratio of the Si‐O‐Si peak height to the ‐OH peak height also decreased as the Fe concentration increased (Figure 5d), suggesting the formation of more FeO(OH) as Fe content increased.

## 3 Discussion

Over 80000 research articles have been published on Si‐based or Si‐releasing materials for orthopedic, bone or tissue engineering applications*, and with some notable clinical and commercial successes (e.g., Actifuse, Bioglass 45S5, BioRoot RCS). The intracellular mechanism for Si‐mediated bone regeneration, however, remains unclear. Here we report that Si (2 mM) inhibited osteoclasts (OC) formation (p<0.0001) in both the primary and RAW264.7 OC subclone (Figures 1 and 2), and we suggest a possible (pre‐transcriptional) mechanism for this Si‐mediated inhibition of osteoclastogenesis by the inhibition of ROS generation via the Fenton reaction.

Other studies have reported Si‐dependent inhibition of osteoclastogenesis, using human CD4+, mouse haematopoietic stem cells, mouse bone marrow, and RAW264.7 cells.^[^ ^9^, ^17^, ^19^, ^30^, ^31^ ^]^ In these studies, different Si concentrations (10 mM‐890 µM) were reported to cause OC inhibition.^[^ ^32^ ^]^ This variation in OC response to Si is likely to be due to differences in the design of the experiments (e.g., cell‐type‐specific responsiveness, seeding number, substrate, RANKL concentrations), source of Si, and outcome measurements (what the authors consider to be functional OCs). Mladenović et al.,^[^ ^18^ ^]^ used a seeding density of 1 × 10^6^/cm^2^ mouse bone marrow cells, whilst Zhou et al., used 1 × 10^5^/cm^2^ human mesenchymal stem cells.^[^ ^33^ ^]^ Here we present the first evidence of Si‐dependent inhibition in both primary and RAW 264.7 OC differentiation and function (resorption activity), together with OC‐specific TRAP‐5b quantification.^[^ ^34^ ^]^

### 3.1 ROS Inhibition of Osteoclastogenesis

We investigated a possible mechanism by which Si, at concentrations relevant to BG‐conditioned media (0.4–2 mM), may cause OC inhibition by modulating ROS availability through iron chelation. Previous studies have demonstrated that intracellular ROS levels are crucial for osteoclastogenesis^[^ ^35^ ^]^ and that the use of antioxidants reduces both osteoclastogenesis^[^ ^36^, ^37^ ^]^ and OC resorption.^[^ ^23^, ^38^ ^]^ Common antioxidants (for example, N‐acetyl cysteine (2.5 mM), ascorbate (0.5 mM), and resveratrol (3 mM)) have been reported to inhibit osteoclastogenesis via a RANKL‐dependent mechanism.^[^ ^39^ ^]^ Furthermore, a mitochondrial‐specific antioxidant (MitoQ 0.25 mM) was reported to be particularly effective in inhibiting the differentiation of RAW264.7 cells to OCs, compared with non‐mitochondrial‐specific antioxidants (N‐acetyl cysteine, RSV, and ascorbate).^[^ ^36^, ^37^ ^]^ In our study, the use of NaPy^[^ ^40^ ^]^ at 10 mM inhibited osteoclastogenesis on day 5 and also appeared to increase mononuclear/macrophage cell proliferation (Figure 2a,c,d). This may be caused by increased pyruvate or pyruvate dehydrogenase kinase, which would enhance the polarisation of M1 macrophages,^[^ ^41^ ^]^ cells that have been shown to inhibit RANKL‐mediated osteogenesis.^[^ ^42^ ^]^

ROS plays an important role in several cellular pathways known to affect osteoclastogenesis, including mitogen‐activated protein kinases (MAPK), NFκB, Ca^2+^, and RANKL.^[^ ^35^, ^39^, ^43^, ^44^, ^45^ ^]^ H_2_O_2,_ for example, has been reported to increase IκBa phosphorylation which subsequently induces the expression of the important osteoclastogenic factor NFκB.^[^ ^46^ ^]^ ROS has also been reported to increase Ca^2+^ release from the endoplasmic reticulum and this increase has been linked to increased gene expression of nuclear factor of activated T‐cells cytoplasmic 1 (NFATc1), a gene important for macrophage fusion into pre‐OCs.^[^ ^43^ ^]^ ROS may also be involved in osteoblast‐OC signaling, where it has been previously reported that the culture of osteoblasts with antioxidants inhibits RANKL production.^[^ ^47^ ^]^ The present work extends these findings by proposing that this Si osteoclast formation inhibition is mediated through suppression of the Fenton reaction, thereby reducing intracellular ROS availability and downstream signaling required for osteoclastogenesis. Notably, our data show that supplementation with FeCl_2_ restores osteoclast formation in Si‐treated cultures, supporting the hypothesis that Si modulates redox‐active iron and thus ROS‐dependent pathways crucial for osteoclast differentiation.

### 3.2 How does Si Reduce ROS Availability?

In our study, we investigated if soluble Si is acting as; 1) an antioxidant that reduces the intracellular ROS level via the release of free H^+^ that reacts with the peroxide (O₂•⁻) or hydronium (H_3_O^+^) via the formation of SiO(OH)_3_^−^ and/or SiO_2_(OH)_2_^2−^ in solution^[^ ^24^ ^]^; or 2) an iron chelator that reduces the activity of the free iron or iron‐containing enzymes (for example, catalase) involved in the Fenton reaction.^[^ ^45^, ^46^ ^]^ The Fenton reaction catalyzes the generation of hydroxyl free radicals (•OH) and hydroperoxyl (HOO•) from peroxide (a vital intercellular reaction). To support his second theory, is known in mining and wastewater treatments that silicate can remove Fe.^[^ ^48^, ^49^ ^]^ Si may therefore maintain the stability of H_2_O_2_ within an iron environment, causing less ROS production.

The decrease in soluble Si and Fe concentrations, when FeCl_2_ and Na_2_SiO_3_ were dissolved together (and filtered), indicates chemical interaction and precipitate formation (Figure 5a,b). The formation of insoluble precipitates was visually confirmed and the presence of iron silicate within the precipitate (Si‐O‐Fe peak ≈675 cm^−1^) was demonstrated via FTIR spectra analyses (Figure 5c). Furthermore, the characteristic ─OH bonds ≈1640cm⁻¹ and ≈1530cm⁻¹ may indicate the presence of FeO(OH) (Figure 5c). Previous studies have shown that the formation of iron silicates is often accompanied by the presence of iron oxyhydroxides.^[^ ^28^ ^]^ The reported Si polymerization above 2 mM, may also increase iron binding capacity^[^ ^50^ ^]^ and further increase aggregation (Figure 5). In our study, we utilized Na₂SiO₃, which in aqueous solution is thought to form orthosilicate species and orthosilicic acid.^[^ ^51^ ^]^ The type of Si species and concentration may also play an important role in modulating cellular responses. Polymerization of silica has been reported to occur at concentrations above ≈2 mM,^[^ ^51^ ^]^ with dimers and higher‐order oligomers potentially influencing cellular interactions through changes in internalization pathways, interactions with biomolecules or ions, and precipitation.

**Figure 6:**
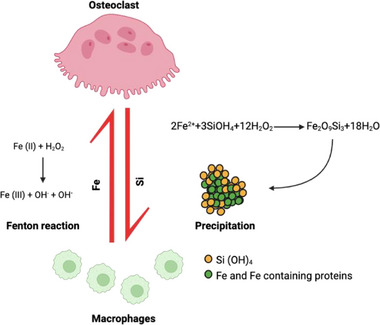
Proposed interaction between Fe and Si and osteoclastogenesis. The chemical interaction of Si with Ferric ions may inhibit the intracellular Fenton reaction, thereby reducing ROS availability and inhibiting osteoclastogenesis. Conversely, the addition of free iron (Fe) increases the availability of redox‐active iron, potentially enhancing the Fenton reaction and restoring osteoclastogenesis.

This interaction between silicate species and redox iron not only promotes aggregate formation but may also influence osteoclast differentiation. We hypothesize that the increased binding capacity of ferric silicate formation could play a crucial role in inhibiting the Fenton reaction within cells, thereby potentially reducing ROS availability and osteoclastogenesis (Figure **6**). The addition of Fe could increase the availability of redox‐active iron, increase ROS availability via the Fenton reaction, and restore osteoclastogenesis (Figure 4). Our findings support a potential Si–Fe interaction mechanism where sodium metasilicate chelates Fe^2^⁺ ions, reducing their participation in the Fenton reaction and lowering intracellular ROS levels. While particle formation was confirmed via FTIR at higher concentrations (1–4 mM), this was not observable at physiologically relevant concentrations used in cell culture conditions, although such interactions may still occur. Fe–Si mineral structures, such as fayalite (Fe₂SiO₄) or siliceous ferrihydrite,^[^ ^52^ ^]^ are known to exist and this further supports the proposed model.

The biochemical assay (Figure 3a) revealed that Si did not reduce ROS availability independently of Fe but did with the addition of Fe (Figure 4). This suggests that our second theory may be correct and that Si inhibits the Fenton reaction. In support of this theory, the addition of Fe to Si restored osteoclastogenesis (Figure 4). Previous studies have also reported that Si dissolution ions from a calcium silicate scaffold (Si 567 mM) reduce ROS production in human bone marrow stem cells.^[^ ^21^ ^]^ And others have reported that the addition of iron (ferric ammonium citrate) could induce OC formation through increased ROS production in both RAW264.7 and bone marrow‐derived macrophages. ^[^ ^38^, ^44^ ^]^ An in vivo study also reported increased serum RANKL levels after administration of 0.04 g kg^−1^ Fe in a mouse model.^[^ ^38^ ^]^

If Si OC inhibition is (in part) acting through an iron chelation pathway, it is also important to note that iron is important in other cellular pathways. TRAP‐5b is an enzyme containing redox‐active Fe and is crucial for ROS‐mediated intracellular transcytosed collagen I degradation (via the Fenton reaction) within bone matrix‐delivering vesicles.^[^ ^53^, ^54^, ^55^ ^]^ Si may therefore inhibit bone resorption (via TRAP‐5b inhibition) in addition to inhibiting osteoclastogenesis. Indeed, Si (1 mM) reduced the volume and depth of resorption pits in dentine (Figure 1b). It is also worth considering that the effect of Si chelation of Fe, is unlikely to be restricted to OCs, and will affect other cells involved in bone fracture repair and remodeling. We observed that Si (> 1.4 mM) reduced ROS levels in osteoblasts (SaOs‐2 cell‐line) (Figure S2, Supporting Information), whilst there was little effect of Si on ROS availability in monocytes (Figure 3b). These findings underscore a complex interplay between silicate polymerization and iron availability, which appears to modulate cellular oxidative processes and influence bone remodeling.

### 3.3 Comparison Between RAW264.7 TRAP+ Subclone and Primary Osteoclasts

Two different OC models (primary and RAW TRAP+ subclone) were used within this paper. While both cells showed inhibition of OC formation in response to Si (2 mM), morphological differences between the OCs formed were observed. The shape differences in OC‐like cells may partly be due to the culture substrate; RAW subclone cells cultured on tissue culture plastic showed more pancake‐like morphology, compared to the bone‐resorbing primary cells cultured on dentine discs. Orriss et al. reported that monocyte contact with a calcium‐containing mineral (and possibly mineralized collagen fibers) is needed for the differentiation of OCs and polarization of primary monocytes, whilst plastic substrates caused a “pancake morphology”, similar to that observed in Figure 2a.^[^ ^56^ ^]^ The high variance observed in RAW246.7 OC size (Figures 2b and 4e) has also been noted by Orriss and Arnett (2012).

RAW264.7 cells offer some practical advantages as an OC model in terms of reproducibility, ease of expansion, speed of differentiation (6 days compared with 9 days from primary cells) and cost (not requiring a macrophage colony‐stimulating factor). The RAW264.7 model, therefore, allows larger experiments and more repeats that are not possible due to the limited number of OCs generated in the primary model. RAW264.7 has been reported to be a heterogeneous population, and the selection of colonies with increased responsiveness to RANKL, increases the relevance of this OC model.^[^ ^57^ ^]^ This is supported by our study, where the RAW264.7 osteoclastic sub‐clone was capable of dentin resorption and expressed Trap‐5b (Figure S1, Supporting Information), and was responsive to both RANKL and Si, whilst the undifferentiation monocytes were not (Figure 3b).

## 4 Conclusion

This paper demonstrated that Si inhibits osteoclastogenesis and that this Si‐mediated OC inhibition is Fe‐dependent. Where Si inhibits ROS production (via the Fenton reaction), and the addition of Fe restores Si‐inhibited osteoclastogenesis. Furthering our understanding of the underlying (and pre‐transcriptional) interactions of Si with OCs will enable the development of optimized Si‐BGs (and other Si ion‐releasing materials), with specific ion release rates customized for specific bone disease applications.

*Search terms:(“silicon releasing” OR “Si releasing” OR “silicon‐releasing” OR “silicon based” OR “silicon‐based” OR “silicon containing” OR “silicon‐containing” OR “silicon dioxide” OR “silicate releasing” OR “silicate‐releasing” OR “silicate based“ OR ”silicate‐based“ OR ”silicate containing“ OR ”silicate‐containing“ OR ”bioactive glass*“ OR ”bioglass*“ OR ”silicate biomaterial*“ OR ”silicate‐based biomaterial*“ OR ”silicate coating*“ OR ”silicon coating*“ OR ”coated with silicate“ OR ”coated with silicon“ OR ”silicon compounds“ OR ”biomaterials“ OR ”silicates“ OR ”bioactive glass“) AND (”bone substitute*“ OR ”bone tissue engineering“ OR ”bone regeneration“ OR ”bone repair“ OR ”osseous substitution“ OR ”bone scaffold*“ OR ”bone biomaterial*“ OR ”bone graft“ OR ”bone implant*“ OR ”orthopedic implant*“ OR ”orthopaedic implant*“ OR ”orthopedic implant* with silicate coating“ OR ”orthopaedic implant* with silicate coating“ OR ”orthopedic implant* with silicon coating“ OR ”orthopaedic implant* with silicon coating“ OR ”bone substitute materials“ OR ”bone implants“ OR ”orthopedic implants“ OR ”tissue engineering“ OR ”bone regeneration“ OR ”bone healing”). The search was conducted on 24th November 2024, Web of Science (all databases).

## 5 Experimental Section

### Cell Culture Models

The cell types used to investigate the role of Si on OCs included primary MF‐1 mouse bone marrow cells, the undifferentiated RAW264.7 cell line, and an osteoclastic subclone of the RAW264.7 cell line. The RAW264.7 macrophage cell line was an in vitro model commonly used for macrophage and OC‐like cells because of the ease of culture, ease of expansion, and short experimental time. There is, however, debate about whether RAW264.7 cells were capable of resorbing bone and were thereby the similarity to primary osteoclasts.^[^ ^57^ ^]^

The RAW 264.7 cell line has been reported to be a heterogeneous cell population containing cells with different RANKL‐sensitivity, some being unresponsive, and others capable of responding to RANKL to form multinucleated TRAP+ OC‐like cells capable of bone resorption.^[^ ^57^ ^]^ A RAW264.7 TRAP+ responsive colony selection approach was adopted, as described by *Cuetara* et al. ^[^ ^57^ ^]^ In brief, the RAW264.7 cells (obtained from ECACC) were expanded in high glucose Dulbecco's Modified Eagle Medium (DMEM) with GlutaMAX (supplemented with 10% (v/v) foetal bovine serum (FBS)), and 50 U ml^−1^ penicillin and 50 µg ml^−1^ streptomycin. RAW OC subclones^[^ ^57^ ^]^ were selected by limited seeding dilution and colony selection with an optimized TRAP‐5b assay. The subclones were then selected and cultured in DMEM GlutaMAX medium, supplemented with 10% FBS and 100 U ml^−1^ penicillin, 100 µg ml^−1^ streptomycin, and 3 ng ml^−1^ RANKL (R&D Systems, UK). The heterogeneous RAW 264.7 cells (without OC differentiation) were used as a macrophage cell line, in comparison to the OC subclone for ROS and VEGF studies, these cells were cultured at 30000/cm^2^.

All experimental osteoclastogenic culture conditions (for example, Si, Fe, sodium pyruvate (NaPy) for both primary and RAW subclones, contained 3 ng ml^−1^ RANKL at a cell seeding of 3000/cm^2^ in 12‐well tissue culture plates (Greiner Bio‐One). Preliminary experiments found that 3 ng ml^−1^ RANKL did not inhibit OC formation (in terms of the number of OCs or resorption capability compared with 20 ng ml^−1^ RANKL). This was more reflective of in vivo RANKL serum levels^[^ ^58^ ^]^ and enabled a model more sensitive to altered culture conditions, compared to 20 ng ml^−1^ RANKL, which was used as a control for maximum OC formation.

### Isolating Primary Mouse Osteoclasts

A primary mouse model was used as previously described by Orriss and Arnett,^[^ ^56^ ^]^ briefly, OC‐forming marrow mononuclear cells were obtained by flushing the long bones of two 6–10‐week‐old MF1 mice. After washing in phosphate‐buffered saline (PBS), marrow cells were initially cultured for 24 h at °C in 5% CO_2_ in 75 cm^2^ flasks (Greiner Bio‐One) in minimal essential medium (MEM) supplemented with 10% FCS, 2 mM L‐glutamine, 100 U ml^−1^ penicillin, 100 µg ml^−1^ streptomycin, and 0.25 µg ml^−1^ amphotericin B containing 200 ng ml^−1^ M‐CSF (R&D Systems). Non‐adherent cells were collected and re‐suspended at 6×10^5^ cells ml^−1^ in supplemented MEM containing 200 ng ml^−1^ M‐CSF and 3 ng ml^−1^ RANKL (R&D Systems), with pH increased to 7.4 by the addition of NaOH. A 200 µl of cell suspension (1.2 × 10^5^ cells) was allowed to sediment for 48 h on to 5 mm diameter dentine discs (elephant ivory kindly donated by Professor Tim Arnett from UCL) in 96‐well plates (Greiner Bio‐One) at 37 °C to allow the attachment of OC precursors. Dentine discs were then transferred to 6‐well plates containing supplemented MEM with 200 ng ml^−1^ M‐CSF and 3 ng ml^−1^ RANKL. Cells were cultured for 7 days in 5% CO_2_ at 37 °C with ion‐conditioned media. For the final 2 days of culture, the medium pH was reduced from 7.4 to 6.8 by the addition of HCl. A disadvantage of the primary mouse model was the limited number of monocytes obtained, which constrains the total number of differentiated OCs obtainable, the experimental size, and repeats. For this reason, quantification of TRAP‐5b was possible only with the RAW264.7 subclone.

### Preparation of Media Containing Si and Fe

Sodium metasilicate (Na_2_SiO_3_, Merck) conditioned media (containing 0.2, 0.4, 1, 1.5, and 2 mM Si) were created, within the [Si] ranges previously reported to be released from bioactive glasses (e.g.,45S5 BG).^[^ ^18^ ^]^ No significant pH change was observed in the Si‐conditioned media. The known ROS scavenger, NaPy was used at a concentration of 10 mM, as preliminary chemical experiments (see 2.5) demonstrated this to have a similar effect on ROS available as 2 mM Si. Iron chloride (FeCl_2_) (Sigma–Aldrich, Merck, UK) was also dissolved in PBS or media at 10 mM as a stock solution before being further diluted to PBS to 10 and 20 mM for an acellular ROS assay or media at 10–100 mM. All conditioned media was filtered through a 0.2 mm syringe filter prior to use.

### Proliferation of Macrophage and Osteoclasts with Si, NaPy, and Fe

Quantification of cell numbers was determined using a DNA quantification kit (Sigma‐Aldrich) following the manufacturer's instructions after 1‐, 2‐, 3‐, and 7‐days cell culture. Briefly, after the removal of media, cells were washed 3 times with PBS. Finally, 200 µl of molecular grade water was added in each well and cells were lysed by 6 times freeze‐thaw cycles, and 50 µl of lysate from each well was transferred to a black optical 96‐well plate. Fluorescence readings were measured (360 nm excitation and 460 nm emission wavelengths) using a fluorescence microplate reader (Fluoroskan Ascent FL, Thermo Labsystems, UK).

### Measurement of ROS Availability in the Presence of Si and Fe

A de‐esterified 2,7‐dichlorofluorescein diacetate (H_2_DCF‐DA) assay was performed using a modified version previously described in PBS.^[^ ^59^ ^]^ Briefly, de‐esterification of 1 mM H_2_DCF‐DA (0.5 ml) was achieved by the addition of 2 ml 10 mM NaOH. Si‐containing samples were pH balanced to 7.2 by the addition of 10 µl 1 M NaOH per 2 ml of solution prior to the addition of 10 or 20 mM FeCl_2_ as a catalyst for the Fenton reaction. After further 5‐min incubation at 37 °C, the solution was transferred to a black optical 96‐well plate, and fluorescence was measured (excitation 495 nm, emission 538 nm) for total ROS generation.

### Intracellular ROS Measurements

Intracellular ROS was detected by 2,7‐dichlorofluorescein diacetate (H_2_DCF‐DA) as previously described.^[^ ^60^ ^]^ Briefly, the total ROS was measured after 3 days’ culture in macrophage‐like RAW264.7 cells, and after 7‐day culture of osteoclastic RAW264.7. Prior to each treatment timepoint, 500 µM H_2_O_2_ was applied directly in the cell culture media and incubated for 30 min. After treatments, media were gently removed, and 200 µl of 10 µM DCFH‐DA (dissolved in warm PBS) was added to each well. The plates were incubated for 30 min at 37 °C in 5% CO_2_. After incubation, the total ROS was measured (as described in acellular measurements).

### TRAP Staining

The number and size of the OCs were determined using a TRAP staining kit, according to the manufacturer's instructions (Sigma Aldrich). Cultures were fixed in 2.5% glutaraldehyde (v/v) for 10 min and washed with PBS. TRAP staining solution was prepared and added to the fixed wells before the samples were incubated for 20 min at 37 °C. After staining, the solution was removed and washed with deionized water before imaging with Nikon brightfield reflected light microscopy with epi‐illumination. OC cells were defined as TRAP+ with more than 3 nuclei. The number of OCs was assessed “blind” to the treatment groups and counted per mm^2^ using a transmitted light microscope (Nikon Labophot 2A microscope, with 100 W epi‐illumination and metallurgical objectives). The size of the OCs was determined by measuring the area of OCs using Image J “freehand selection” and measurement function with a prior setting of global scale.^[^ ^56^ ^]^

### Quantification of TRAP‐5b Enzyme Activity

RAW264.7 OC subclone cells were seeded in 6‐well plates at a concentration of 3 × 10^3^ cells cm^−2^ in DMEM + GlutaMAX with the addition of RANKL (3 or 20 ng ml^−1^). The cell lysates (produced by freeze‐thaw cycles) were incubated at 37 °C for 2 h together with TRAP assay reagent in a transparent clear 96‐well plate. The reaction was stopped after 2 h by adding 1 M NaOH. TRAP‐5b activity was quantified by absorbance measurements at 405 nm (Tecan Infinite 200 PRO microplate reader).

### Quantification of Osteoclast Resorption

OC resorption was determined using an interferometer (Zygo NewView 200 3D optical interferometer) following culture on dentine discs. After 11 days OCs and dentine discs were fixed with 2.5% glutaraldehyde and the total resorption area per mm^2^ and average resorption pit depth in µm (1×0.25mm^2^ areas sampled in 15 different samples for each treatment) determined with the MetroPro application.

### Evaluation of Resorption Morphology by Scanning Electron Microscopy (SEM)

To confirm OC formation and resorption capability (on dentin discs), SEM was performed following protocols reported by Turmaine et al.^[^ ^61^ ^]^ Briefly, OC cultures were fixed in 2% paraformaldehyde (w/v) and 1.5% glutaraldehyde (v/v) in a 0.1 M sodium cacodylate buffer (pH 7.3) for 24 h at 3 °C. The fixed dentine discs were then washed twice in 0.1 M sodium cacodylate, followed by 30 min incubation at room temperature. A 1% solution of osmium tetroxide and 1.5% potassium ferrocyanide in 0.1 M cacodylate buffer at 3 °C was added and incubated at room temperature for 90 min. The samples were washed in 0.1 M cacodylate buffer followed by distilled water and then dehydrated in a graded ethanol‐water series (50, 75, 95, and 100% ethanol), followed by critical point drying using a CO_2_ chamber mounted on aluminium stubs with carbon tape. The mounted samples were coated with a ≈2 nm layer of palladium using a Gatan ion beam coater. The images were viewed with a Jeol 7401 field emission gun – scanning electron microscope (FEG‐SEM).

### Characterization of Si and Fe Precipitate Properties

To investigate the potential precipitation of Si and Fe, solutions containing 2 mM Si with varying FeCl₂ concentrations (0.1 to 4 mM) were prepared in water and incubated overnight at room temperature. The solutions were filtered using a 0.2 µm syringe filter to remove precipitates, which were analyzed using ATR‐FTIR (Jasco FT/IR‐4200), while the soluble Si and Fe concentrations in the filtrate were measured using inductively coupled plasma optical emission spectroscopy (ICP‐OES, Thermo iCAP 6300). Precipitates were collected by freezing the Si and Fe solutions, followed by freeze‐drying using a FreeZone 2.5 Litre ‐50 °C Benchtop freeze dryer. The ATR‐FTIR spectra were processed using OriginPro, with the baseline subtracted via the line interpolation method and smoothened using the Savitzky‐Golay method with a second‐order polynomial. The spectra were normalized to (0,1) to enable comparison of peak heights.

### Statistical Analysis

Experimental data were presented as mean ± standard deviation (SD). Data normality was evaluated using a Q–Q test. For normally distributed data, group differences were analyzed using one‐way analysis of variance (ANOVA) followed by Tukey's post‐hoc test for multiple comparisons. Non‐normally distributed data were analyzed using the Kruskal–Wallis followed by Dunn's post hoc test. Statistical significance was defined as p < 0.05, with significance levels indicated as follows: *p < 0.05, **p < 0.01, and ***p < 0.001.

## Conflict of Interest

The authors declare no conflict of interest.

## Author Contributions

Y.L. performed in writing–original draft, data analysis, data curation, methodologies, visualization. A.‐M. R. performed in writing–original draft, data analysis, data curation, methodologies, visualization. A.R. performed in writing–review and editing. J.T. performed in writing–review and editing, data curation, and data analysis. K.S. performed in writing–review and editing. A.O. performed in writing–review and editing, validation. T.K. performed in writing–review and editing, validation. G.J. performed in writing–review and editing, writing–, project administration, investigation, formal analysis, data curation, and conceptualization.

## Supporting information



Supporting Information

## Data Availability

The data that support the findings of this study are available from the corresponding author upon reasonable request.
