# Supplementary material for: Si Inhibited Osteoclastogenesis: The Role of Fe and the Fenton Reaction
Source: Adv Healthc Mater. 2025 Jun 5;14(31):2501086. doi: 10.1002/adhm.202501086 (PMC12683221; doi:10.1002/adhm.202501086)
Supplement: Supplementary file 1 — Supporting Information [file ADHM-14-0-s001.pdf]

ADVANCED HEALTHCARE MATERIALS

Supporting Information

for *Adv. Healthcare Mater.*, DOI 10.1002/adhm.202501086

Si Inhibited Osteoclastogenesis: The Role of Fe and the Fenton Reaction

*Yutong Li, Adriana-Monica Radu, Azadeh Rezaei, Joel Turner, Kaveh Shakib, Akiko Obata, Toshihiro Kasuga and Gavin Jell**

Supporting Information

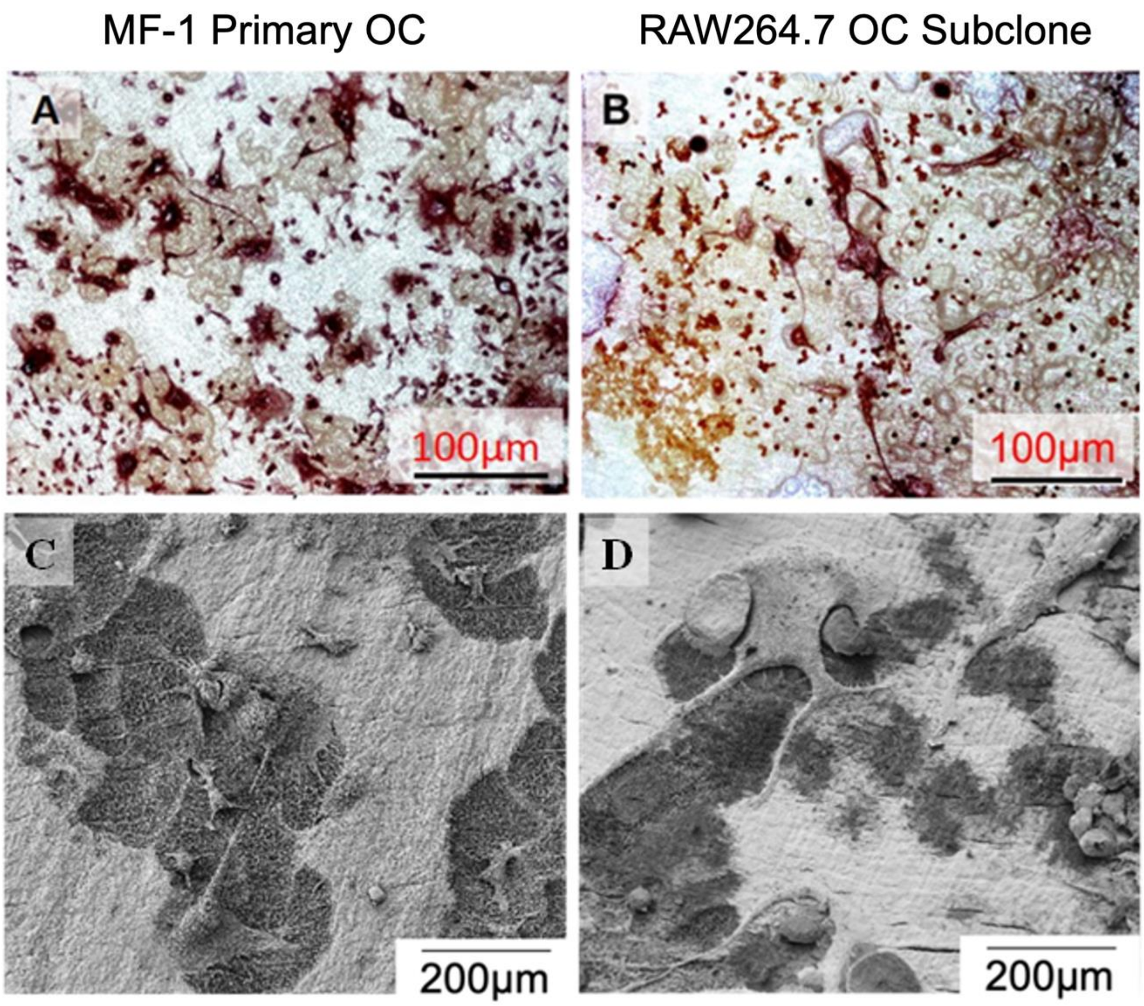

Figure S1. Osteoclast (OC) formation from primary mouse cells and a subclone derived from the RAW264.7 macrophage cell line. Primary OCs were generated from MF-1 mouse bone marrow cells and cultured on dentine discs for 9 days. TRAP staining and resorption pits were visualized using reflected light microscopy (A). Pre-osteoclasts derived from the RAW264.7 cell line were isolated via limiting dilution and assessed for TRAP expression and resorptive activity. TRAP staining and resorption pits were observed using reflected light microscopy (B). Scanning electron microscopy (SEM) on dentine (C) revealed resorption patterns and collagen fiber exposure in both cell types (primary OCs (C), RAW264.7 subclone, (D)).

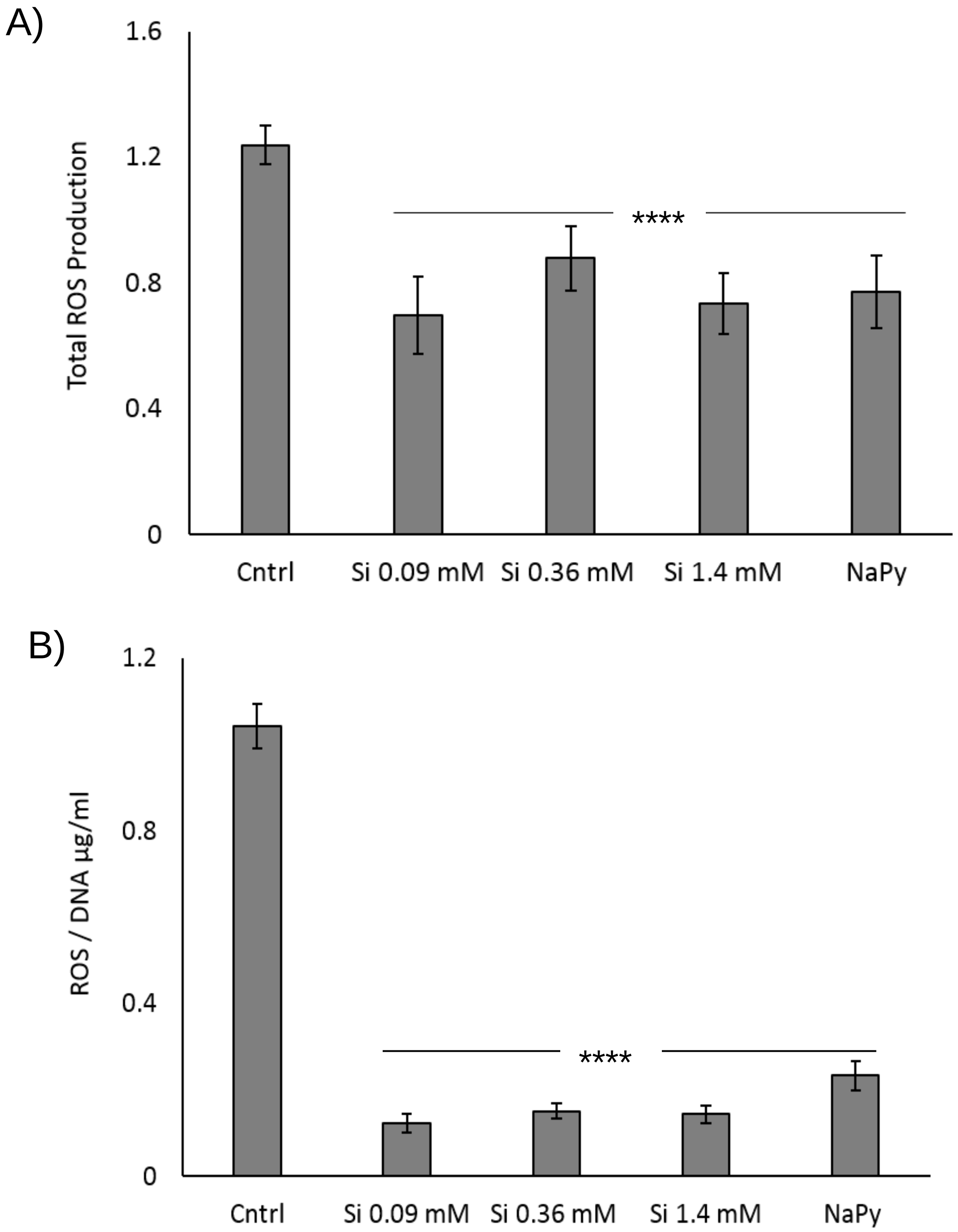

Figure S2. Reactive oxygen species (ROS) production by SaOs-2 cells over 7 days. Total ROS production by SaOs-2 cells with clinically relevant concentrations of soluble silicates are shown in A), along with ROS normalized to DNA content, indicating significant inhibition by soluble silicate (Si) at concentrations below 1.4 mM. In comparison, ROS levels in sodium Pyruvate (NaPy) cultures were significantly higher (B).
